# Increasing Phosphatidylinositol (4,5)-Bisphosphate Biosynthesis Affects Basal Signaling and Chloroplast Metabolism in *Arabidopsis thaliana*

**DOI:** 10.3390/plants3010027

**Published:** 2014-01-03

**Authors:** Yang Ju Im, Caroline M. Smith, Brian Q. Phillippy, Deserah Strand, David M. Kramer, Amy M. Grunden, Wendy F. Boss

**Affiliations:** 1 Department of Plant and Microbial Biology, North Carolina State University, Raleigh, NC 27695, USA; E-Mails: yangju92@hotmail.com (Y.J.I.); cmsmith5@ncsu.edu (C.M.S.); brian_phillippy@ncsu.edu (B.Q.P.); amy_grunden@ncsu.edu (A.M.G.); 2 DOE-Plant Research Laboratory, Michigan State University, East Lansing, MI 48824, USA; E-Mails: strandd1@msu.edu (D.S.); kramer8@msu.edu (D.M.K.)

**Keywords:** phosphoinositide, inositol trisphosphate, phosphatidylinositol phosphate kinase, chloroplast, starch, carbon metabolism, photosynthesis, calcium, *Arabidopsis*

## Abstract

One challenge in studying the second messenger inositol(1,4,5)-trisphosphate (InsP_3_) is that it is present in very low amounts and increases only transiently in response to stimuli. To identify events downstream of InsP_3_, we generated transgenic plants constitutively expressing the high specific activity, human phosphatidylinositol 4-phosphate 5-kinase Iα (*Hs*PIPKIα). PIP5K is the enzyme that synthesizes phosphatidylinositol (4,5)-bisphosphate (PtdIns(4,5)P_2_); this reaction is flux limiting in InsP_3_ biosynthesis in plants. Plasma membranes from transgenic *Arabidopsis* expressing *Hs*PIPKIα had 2–3 fold higher PIP5K specific activity, and basal InsP_3_ levels in seedlings and leaves were >2-fold higher than wild type. Although there was no significant difference in photosynthetic electron transport, *Hs*PIPKIα plants had significantly higher starch (2–4 fold) and 20% higher anthocyanin compared to controls. Starch content was higher both during the day and at the end of dark period. In addition, transcripts of genes involved in starch metabolism such as SEX1 (glucan water dikinase) and SEX4 (phosphoglucan phosphatase), DBE (debranching enzyme), MEX1 (maltose transporter), APL3 (ADP-glucose pyrophosphorylase) and glucose-6-phosphate transporter (Glc6PT) were up-regulated in the *Hs*PIPKIα plants. Our results reveal that increasing the phosphoinositide (PI) pathway affects chloroplast carbon metabolism and suggest that InsP_3_ is one component of an inter-organelle signaling network regulating chloroplast metabolism.

## 1. Introduction

The phosphoinositide (PI) pathway, which includes inositol phospholipids and inositol phosphates, is implicated in many aspects of plant biology including vesicle trafficking [1,2,3], tip growth [4,5,6,7,8], receptor regulation [9,10,11], light signaling [12,13], stomatal pore regulation [14,15,16], sugar sensing [17], symbiosis [18,19] and protein turnover [20,21]. In the canonical pathway, phosphatidylinositol (4,5) bisphosphate (PtdInsP_2_) is hydrolyzed by phospholipase C (PLC) to generate inositol (1,4,5) trisphosphate (InsP_3_). PtdInsP_2_ also can be dephosphorylated by a 5-phosphatase (ptase) to produce PtdIns4P, which is critical for membrane trafficking and root growth [22,23]. Investigations into pathway function by altering expression of selective genes have led to important insights as to the functions of the proteins and metabolites in plant signaling [24,25,26,27,28]. However, because signaling metabolites by nature are rapid and transient, it has been difficult to identify events downstream of InsP_3_ or InsP_3_-mediated responses. The term InsP_3_-mediated is used to denote all events downstream of InsP_3_ (*i.e.*, InsP_4_, InsP_5_, InsP_6_ and InsP_(7/8)_-mediated signaling). In animal cells, cytosolic InsP_3_-mediated signaling has been shown to contribute to basal mitochondrial metabolism by affecting the activity of calcium-regulated tricarboxylic acid cycle enzymes [29]. By mutating the ER InsP_3_ receptor and thus eliminating basal InsP_3_-mediated increases in cytosolic calcium, the authors found that InsP_3_-mediated release of calcium from the ER was essential for optimal mitochondrial function. These studies and others in *Drosophila* [30] revealed that InsP_3_ contributed to the coordination of inter-organelle metabolism in non-stimulated cells. The role of cytosol InsP_3_ coordinating inter-organelle metabolism has not been investigated in plants. 

Fluctuations in cytosolic calcium occur in the light and dark and have a circadian rhythm [31,32,33,34,35]. Furthermore, in plants, the chloroplast is a major store of intracellular calcium and stromal calcium has been reported to change with light/dark transitions [35,36,37]. Chloroplast stromal calcium is low in the light and increases transiently for about 20 min at the end of day/beginning of dark. Photosynthetic electron transport is not required for dark-induced stromal calcium changes suggesting that proton motive force is not essential for the stromal calcium increase during the light/dark transition [37]. The transient increase in stromal calcium in the dark has been proposed to contribute to the down regulation of Calvin-Benson cycle enzymes such as fructose 1,5-bisphosphatase (FBPase) and seduloheptulose 1,7-bisphosphatase (SBPase) and to the dark deactivation of the ATP synthase [36,38,39,40]. While there are several reports indicating a role for chloroplast calcium and changes in stromal calcium during the light/dark transition, the role of cytosolic calcium in regulating chloroplast metabolism remains a conundrum [40,41]. 

The earliest evidence for a role of the PI pathway and light signaling was from the work of Ruth Satter’s laboratory using *Samanea samman* pulvini [42]. Subsequently, it was shown that increases in InsP_3_ were associated with light-induced shrinking of flexor cells [43]. More recently, blue light signaling was correlated with changes in InsP_3_ in *Arabidopsis* seedlings [12]. Notably, Chen *et al*. [12], found that in *Arabidopsis* seedlings, InsP_3_ was higher in wild type seedlings in the light relative to the dark. Additional evidence that changes in InsP_3_ correlate positively with light/dark transitions comes from two studies. In C4 plants, phosphoenolpyruvate phosphate carboxylase (PEPC) is activated in the light by phosphorylation by PEPC kinase. Coursol *et al*. [44] showed that an increase in InsP_3_ preceded the increase in PEPC kinase activity. In a separate study, *Arabidopsis* plants with mutation in sac9, a PtdInsP_2_ ptase, had increased InsP_3_ [45] and were identified in a screen for plants with a delay in dark adapted deactivation of the ATP synthase [46]. These studies suggest that fluctuations in InsP_3_ could contribute to light/dark regulation in the chloroplast. 

It is difficult to identify events downstream of InsP_3_ *in planta*. One approach that has been used is to remove or dampen the InsP_3_ signal. Perera *et al*. [47] expressed the more active human InsP 5-ptase and lowered basal InsP_3_ in *Arabidopsis* plants. These InsP 5-ptase transgenic plants revealed that InsP_3_-mediated responses were a component of gravitational signaling (the gravitational response in both roots and shoots was delayed) and contributed to about 30% of the stimulus-induced cytosolic, aequorin-sensitive calcium signal in response to salt or cold [15]. While dampening the InsP_3_ signal revealed a decrease in response to gravity attributable to InsP_3_, the targets of InsP_3_-mediated signaling were not identified and the effects of InsP_3_ on plant responses have been questioned [48]. 

In this work, to identify InsP_3_-mediated events, we increased the biosynthesis of InsP_3_. Our approach was to increase the synthesis of PtdInsP_2_, the flux limiting step in plant PI metabolism [49], by expressing a green fluorescent protein (GFP)-fusion construct of the human phosphatidylinositol phosphate 5-kinase1α (*HsPIPKI*α) in *Arabidopsis* plants. *Hs*PIPKIα has a lower Km for PtdInsP and a higher Vmax making it more effective than the *Arabidopsis* PIPKs [50]. Plants expressing the *Hs*PIPKIα had more than 2-fold increased PtdInsP_2_ and InsP_3_ in the leaves. There was a 10% decrease in total calcium suggesting a net efflux of calcium in response to increased InsP_3_ as was found when *Hs*PIPKIα was expressed in tobacco cells grown in suspension culture [49] suggesting that the InsP_3_-sensitive component of the organelle mobile calcium stores might be depleted in these cells. We found the *Hs*PIPKIα expressing plants have higher starch both at the end of day and end of night suggesting decrease in transitory starch turnover and delay in the dark adaptation of the Calvin-Benson cycle. In addition, the *Hs*PIPKIα plants were drought sensitive, but seedlings were more heat and light tolerant than the controls. While at first this seems counter intuitive, *i.e.*, higher cytosolic InsP_3_ should increase calcium signaling, it is possible that the constitutively increasing InsP_3_ in the cytosol decreased the stores of cellular calcium and decreased or delayed dark adaptation and responses to other environmental cues. In summary, we demonstrate that increasing the flux through the PI pathway in plants affects chloroplast carbon metabolism and plant responses to environmental stress, and we hypothesize that InsP_3_-mediated signaling contributes to coordinating inter-organelle metabolism in plants. Future studies monitoring organelle calcium are needed to test this hypothesis. 

## 2. Results and Discussion

### 2.1. Generation and Growth of HsPIPKIα Transgenic Plants

**Figure 1:**
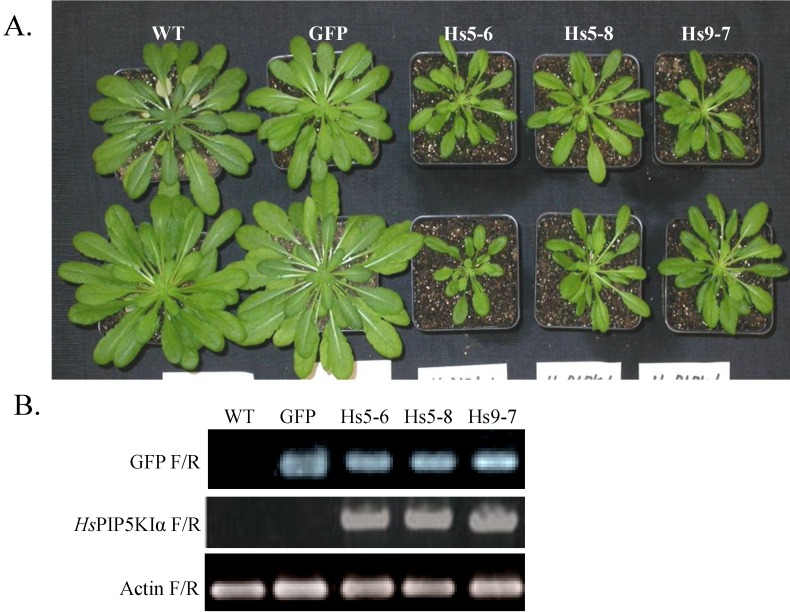
*Arabidopsis* plants expressing GFP-*Hs*PIPKIα (Hs5-6, Hs5-8, Hs9-7) have smaller leaves compared to control plants (WT and GFP-expressing plants). (**A**) Plants were grown under short-d conditions (8 h light/ 16 h dark) for 6 weeks; (**B**) Expression of GFP-*Hs*PIPKIα in 14-day-old transgenic *Arabidopsis* plants is shown by RT-PCR analysis using internal GFP primers and *Hs*PIPKIα specific primers to detect the transcript. Primers specific for *Arabidopsis* actin were used for the loading control.

Three independent transgenic *Arabidopsis* lines carrying the GFP fused human *HsPIPKI*α construct under the control of the cauliflower mosaic virus 35S promoter were generated as described by Im *et al.* [49] by *Agrobacterium*-mediated transformation using vacuum infiltration. GFP-*Hs*PIPKIα plants (hereafter noted as *Hs*PIPKIα plants) are smaller than WT and GFP alone under normal short-day growth condition (8 h of light/16 h of dark) (Figure 1A). Transcripts were confirmed by reverse transcription (RT)-PCR using internal *GFP* forward and reverse primers and *Hs*PIPKIα forward and reverse primers (Figure 1B). No transcript was detected in the wild type using both primer sets. GFP transcripts were detected in GFP expressing lines (GFP alone and the *Hs*PIPKIα lines). 

**Figure 2:**
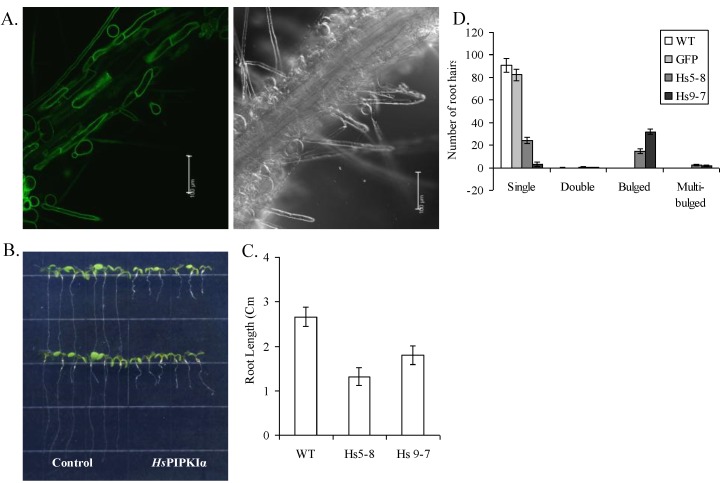
*Arabidopsis* plants expressing GFP-*Hs*PIPKIα (Hs5-6, Hs5-8, Hs9-7) have shorter roots than the control plants (WT and GFP expressing plants). (**A**) GFP-*Hs*PIPKIα localized with the plasma membrane in the root. Transgenic *Hs*PIPKIα seedlings were imaged using a confocal microscope. Left panel shows fluorescence and right panel shows the differential interference contrast image. Bars = 100 μm; (**B**) 10-d-old *Hs*PIPKIα seedlings grown on MS media have significantly shorter roots; (**C**). Plates were photographed and root growth was measured using Adobe Photoshop and analyzed using Microsoft Excel. The data are the mean of at least 10 seedling measurements per line ± SD. (**D**) The number of single, double, bulged and multibulged root hairs in 7-d-old wild-type and *Hs*PIPKIα seedlings were determined. The data are means of 24 seedlings per line ± SE.

In the *Hs*PIP5KIα plants, GFP fluorescence was localized at the plasma membrane (Figure 2A). *Hs*PIPKIα seedlings have shorter roots and stunted and bulged root hairs compared to WT and GFP plants (Figure 2B,C). Seedlings were grown under short-day cycle in MS media. The different types of root hairs were counted for WT, GFP and two independent transgenic lines, *Hs*PIPK5-8 and *Hs*PIPK9-7 when seedlings were 6 days old (Figure 2D). A similar root hair phenotype has been described by others overexpressing the plant PIPKs *in planta* and has been associated with defects in vesicle trafficking and cell wall biosynthesis in tip growing cells [2,4,6,51]. 

### 2.2. PtdInsP_2_ and PIPK Specific Activity Increased in Seedlings and Leaves

**Figure 3:**
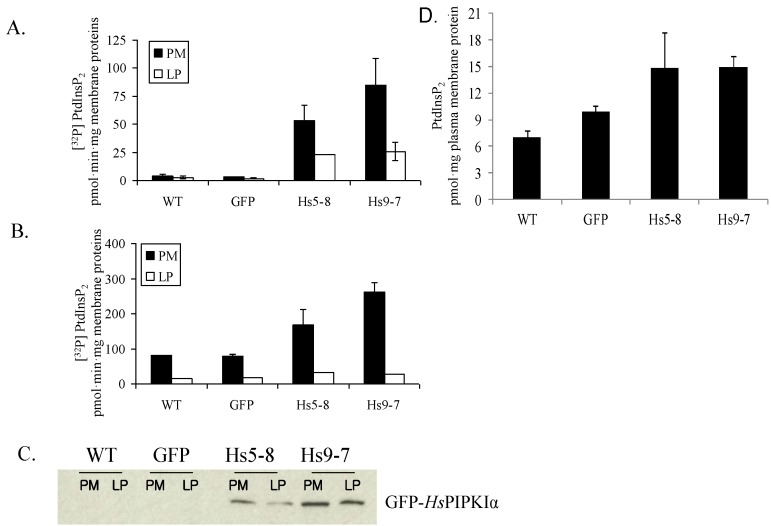
Membrane-associated PtdInsP 5-kinase specific activity from 17-d-old seedlings (**A**) and leaves from 1 month-old plants (**B**); The plasma membrane (PM) and lower phase fraction (LP) from wild-type and transgenic plants were separated by aqueous two-phase partitioning and analyzed for PtdInsP 5-kinase activity with added substrate, PtdIns(4)P. The data are the mean of duplicate values ± SD; (**C**) Immunoblot of proteins from PM or LP of leaves from 1-month-old plants visualized using an antibody to GFP; (**D**) Mass measurement of PtdInsP_2_ based on head group analysis of lipids extracted from isolated plasma membranes of leaves from the 1-month-old plants.

The *Hs*PIPKIα 9–7 lines had a more pronounced bulged root hair phenotype (Figure 2D) and the highest PIP 5-kinase activity (Figure 3A). Plasma membranes were isolated from young seedlings and leaves of 1 month-old plants, and PtdInsP 5-kinase specific activities were measured *in vitro*. Exogenous PtdIns(4)P was added to the reaction mixture so that the substrate would not be limiting. Expression of the *Hs*PIPKIα in *Arabidopsis* increased the production of [^32^P]PtdIns(4,5)P_2_ 15 to 25-fold more in young seedlings and 2 to 3-fold more in 1 month-old plants compared to WT and GFP lines (Figure 3A,B). As indicated by the *in vitro* assays (Figure 3A) and immunoblot of isolated proteins (Figure 3C), GFP-*Hs*PIPKIα was recovered primarily in the plasma membrane (upper phase) that was separated by aqueous two-phase partitioning. 

Head group analysis was used to measure the endogenous PtdInsP_2_. For these experiments, lipids were extracted from plasma membranes of transgenic and control seedlings and the inositol head group was hydrolyzed with HCl. The total Ins(1,4,5)P_3_ released was measured using an Ins(1,4,5)P_3_ assay kit. The transgenic plants had 2 to 2.5-fold increased PtdIns(4,5)P_2_ compared to WT and GFP plants (Figure 3D). 

**Figure 4:**
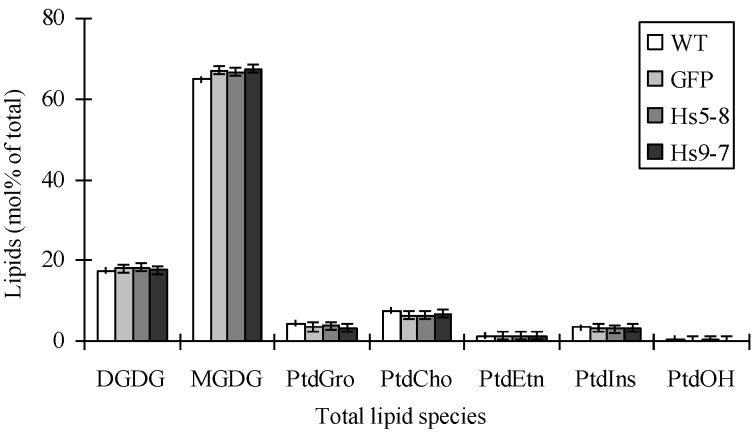
Polar lipid classes (mol % of total polar glycerolipids analyzed) in 3 week-old seedlings from wild-type, GFP and HsPIPKIαlines. The major phospholipid classes (phosphatidylcholine [PtdCho], phosphatidylethanolamine [PtdEtn], phosphatidylglycerol [PtdGro], and phosphatidylinositol [PtdIns]), galactolipid classes (monogalactosyldiacylglycerol [MGDG] and digalactosyldiacylglycerol [DGDG]), and minor phospholipid classes (phosphatidylserine [PtdSer] and phosphatidic acid [PtdOH]) were present. Values are average ± SD (n = 5).

In order to determine if the increased PtdIns(4,5)P_2_ changed the major phospholipids in *Hs*PIPKIα seedlings, total lipids were extracted as described in the Experimental Section. The major composition of the phospholipids, such as PtdGro, PtdEtn, PtdIns, PtdCho, PtdSer and PtdOH, and galactolipids, such as MGDG and DGDG, was not significantly different between WT, GFP and *Hs*PIPKIα plants (Figure 4; the data are presented in Supplemental Data File 1). 

### 2.3. Increased Flux through the Phosphoinositide Pathway in HsPIPKIα Transgenic Plants

**Figure 5:**
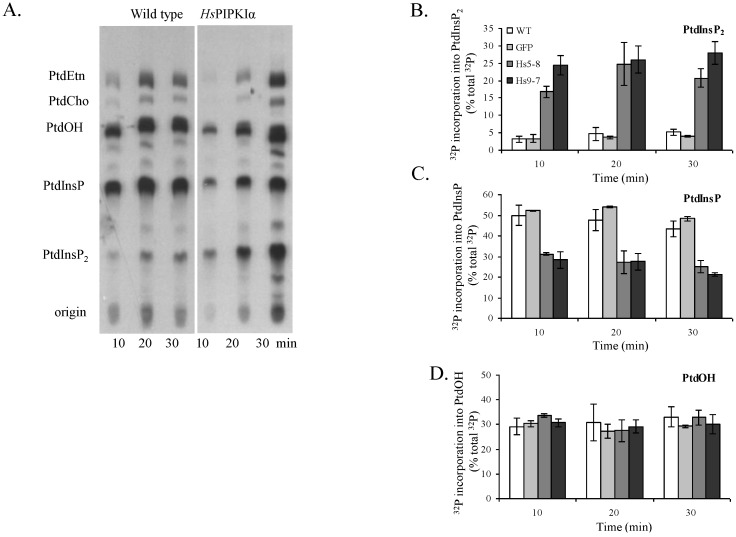
*In vivo* labeling studies with ^32^Pi indicate a rapid rate of [^32^P]PtdInsP_2_ biosynthesis in the *Hs*PIPKIα lines. 13 or 17-d-old seedlings were pre-equilibrated in MS medium overnight; ^32^Pi (50 μCi per sample) was added, the seedlings were harvested, and lipids were extracted at the time points indicated. The lipids were separated by TLC, and ^32^P-labeled lipids were quantified with a Bioscan imaging scanner. (**A**) Representative autoradiogram of the TLC plate; (**B**, **C**, and **D**) show ^32^P recovered phospholipids (PtdInsP_2_, PtdInsP, and PtdOH, respectively) over the time course. The data are reported as percentage of total cpm recovered per lane. Each point is the average ± SD of duplicates from two or three independent experiments except for GFP which is the average ± SD of duplicates from one experiment.

To monitor the rate of PtdIns(4,5)P_2_ biosynthesis *in vivo*, we labeled the seedlings with ^32^Pi and harvested at each time point indicated (Figure 5A). Lipids were extracted and separated by thin layer chromatography (TLC). The incorporation of ^32^Pi into PtdIns(4,5)P_2_ was 5 to 7-fold higher in *Hs*PIPKIα plants compared to WT and GFP plants and saturated by 20 min when calculated as total [^32^P]-labeled lipids (Figure 5B). The incorporation of ^32^Pi into PtdInsP was ~40% less in *Hs*PIPKIα plants compared to WT and GFP plants. This is likely a result of the fast conversion of PtdIns(4)P to PtdIns(4,5)P_2_ in *Hs*PIPKIα plants (Figure 5C). We also labeled the seedlings with [^3^H]*myo*-inositol for 4 days to monitor the levels of intermediates of the PI pathway. In the WT, the ratio of total cellular [^3^H]PtdIns(4)P to [^3^H]PtdIns(4,5)P_2_ was ≥20:1, whereas the ratio was reduced to 2:1 in the *Hs*PIPKIα plants (Table 1). Note there was ~20% decrease in [^3^H]PtdIns(4)P in *Hs*PIPKIα plants which would be anticipated with an increase in PtdIns(4,5)P_2_ biosynthesis. The data are consistent with previous work indicating that PIPK activity is a flux-limiting step in the plant PI pathway [49,52].

**Table 1:** [^3^H]*myo*-inositol labeled PtdInsP_2_ increased in the *HsPIPKIα* plants. Seedlings were incubated with [^3^H]*myo*-inositol, inositol lipids were extracted, separated by thin layer chromatography and quantified using a Bioscan Imaging Scanner. Data are reported as % total [^3^H] inositol lipid recovered. The data are the mean ± SD of 6 values from two biological replicates.

Plant type	PtdInsP_2_	PtdInsP	PtdIns	Ratio of PtdInsP/PtdInsP_2_
WT	0.3 ± 0.2	7.0 ± 0.3	31.9 ± 1	22
*Hs*PIPKIα 9-7	2.9 ± 0.1	5.9 ± 0.2	37.4 ± 1	2

**Figure 6:**
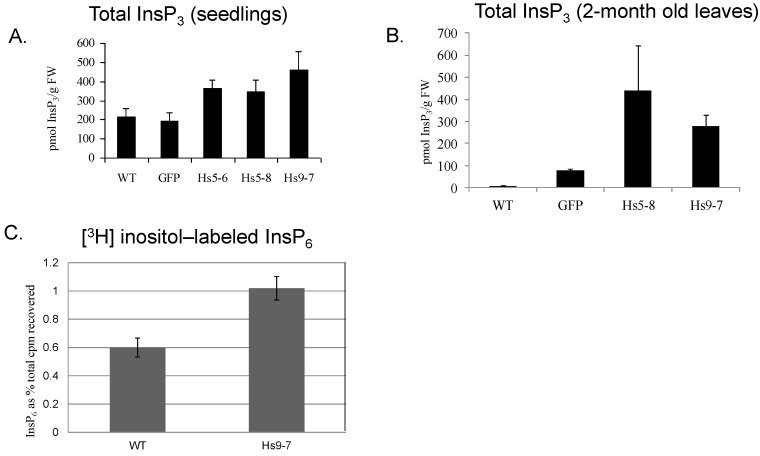
InsP_3_ increased in the *Hs*PIPKIα plants. Based on mass measurement, basal Ins(1,4,5)P_3_ is higher in the 2 week-old *Hs*PIPKIα seedlings (**A**) and in leaves of 2 month-old *Hs*PIPKIα plants harvested in the afternoon (**B**); [^3^H]*myo*-inositol labeling of seedlings indicates increased [^3^H] InsP_6_ production (**C**); The data are the mean ± SD of duplicate samples from two biological replicates.

To determine how increased PtdIns(4,5)P_2_ levels would affect the total cellular Ins(1,4,5)P_3_ levels, we measured the total Ins(1,4,5)P_3_ in the soluble fraction of WT and *HsPIPKI*α plants using an Ins(1,4,5)P_3_ assay kit (Figure 6A). The basal Ins(1,4,5)P_3_ levels were increased 2 to 4-fold in the seedlings and in leaves of 2-month-old plants of *Hs*PIPKIα compared to WT and GFP plants (Figure 6B). These data combined with the radioisotope labeling data indicate that the *Hs*PIPKIα plants had increased flux in the PI pathway and were producing more InsP_3_. 

**Figure 7:**
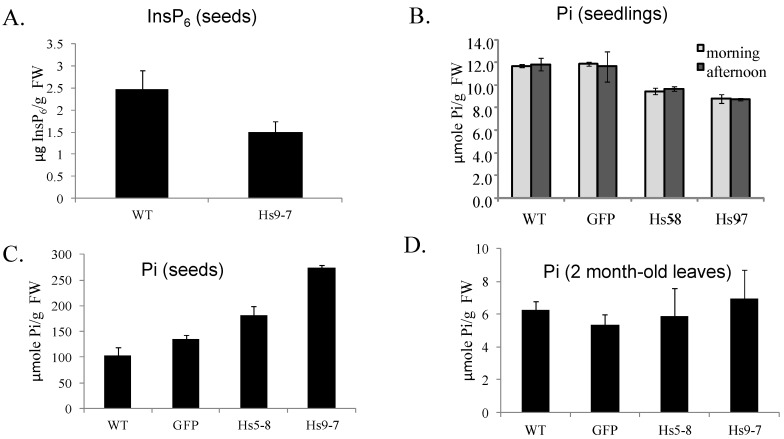
Total InsP_6_ decreased (**A**) and HOAc-soluble Pi (**B**) increased in *Hs*PIPKIα seeds. The InsP_6_ data are the means ± SD of triplicate biological samples. The Pi data are the means of duplicate samples ± SD from 2 independent experiments. In seedlings, the HOAc-soluble Pi decreased by about 20% in the *Hs*PIPKIα lines compared to wild type (**C**) There was no significant difference in HOAc-soluble Pi from 2 month-old leaves; (**D**) The data in (**C**) are the mean ± SD of 3 biological replicates harvested before the lights come on (morning) or before dark (afternoon). The data in (**D)** are the mean of duplicate samples ± SD from 2 independent experiments.

While InsP_3_ can generate calcium oscillations *in vivo*, it can also produce higher ordered InsPs [26,53,54]. We monitored the production of InsP_6_ using both isotope labeling and mass measurement. The [^3^H]*myo*-inositol labeling of seedlings revealed a significant increase in [^3^H]-labeled InsP_6_ indicating that the increase in InsP_3_ had affected higher ordered InsP biosynthesis (Figure 6C). To assess total InsP_6_, we used isocratic ion chromatography as described in the Experimental Section. In a preliminary experiment, we did not detect differences in total InsP_6_ in seedlings (data not shown). Because it was difficult to obtain enough material to do InsP_6_ mass measurements on the seedlings, we analyzed seeds (Figure 7A). InsP_6_ is produced by two pathways: a lipid-mediated pathway resulting from the phosphorylation of lipid-generated Ins(1,4,5)P_3_ and a non-lipid-dependent pathway, which involves *de novo* synthesis and the sequential phosphorylation of *myo*-1L-inositol phosphate [53]. The non-lipid-dependent pathway is the dominant pathway in storage tissue [55] and as shown in Figure 7A, the seeds from the *Hs*PIPK1α plants had 40% less total InsP_6_. Typically seeds with low InsP_6_ have high Pi [56,57] and this is what we found for the *Hs*PIPK1α. The seed HOAc-soluble Pi in the *Hs*PIPK1α lines was almost twice that of the controls (Figure 7B). In contrast, the seedling HOAc-soluble Pi was about 20% less than wild type (Figure 7C) and there was no significant difference in HOAc-soluble Pi in 2-month-old mature leaves (Figure 7D). These data suggest that down regulation of the non-lipid-dependent pathway was compensating for the increased flux through the PI pathway in seedlings and leaves, and that in seeds where the non-lipid pathway was dominant, down regulation led to a net decrease in InsP_6_. More extensive flux analyses of both the lipid- and non-lipid mediated pathway for InsP_6_ biosynthesis are needed in order to determine whether the non-lipid pathway is down regulated in the leaves of the *Hs*PIPK1α plants.

Analysis of the seedlings using inductively coupled plasma (ICP) indicated that the total Pi, calcium and magnesium were slightly lower in the *Hs*PIPKIα transgenics compared to controls (Table 2). The decrease in total calcium would be anticipated if the increased flux through the PI pathway resulted in a constitutive signal such that there is a net efflux of calcium from the cells [49,58]. 

**Table 2:** Calcium, phosphorus and magnesium are lower in the shoots of 3 week-old *Hs*PIPKIα seedlings. The samples were analyzed on a Perkin Elmer inductively coupled plasma-optical emission spectrometer (ICP-OES). The data are means ± SE from three independent experiments.

Plant	Concentration (mg/dry weight (g))						
	P	Ca	K	Mg	S	Mn	Fe
WT	9.5 ± 0.1	5.8 ± 0.1	60.5 ± 0.6	2.5 ± 0.04	11.1 ±0.8	0.2 ± 0.003	1.0 ± 0.3
GFP	9.0 ± 0.5	5.1 ± 0.1	58.2 ± 0.6	2.2 ± 0.04	9.7 ± 0.5	0.2 ± 0.005	1.2 ± 0.2
Hs5-8	8.6 ± 0.1	4.2 ± 0.1	61.2 ± 0.3	1.7 ± 0.04	8.8 ± 0.6	0.2 ± 0.006	0.9 ± 0.2
Hs9-7	7.8 ± 0.4	4.5 ± 0.1	61.1 ± 1.3	1.8 ± 0.04	9.8 ± 0.9	0.2 ± 0.005	0.9 ± 0.3

### 2.4. Starch Metabolism Is Altered HsPIPKIα Plants

In all plants, the PIP 5-kinase specific activity was higher in the leaves of the older plants compared to the seedlings and InsP_3_ was higher in mature leaves in the afternoon versus morning. For these reasons and because there appeared to be less effect on leaf morphology than root morphology, we focused our studies on leaf metabolism. 

**Figure 8:**
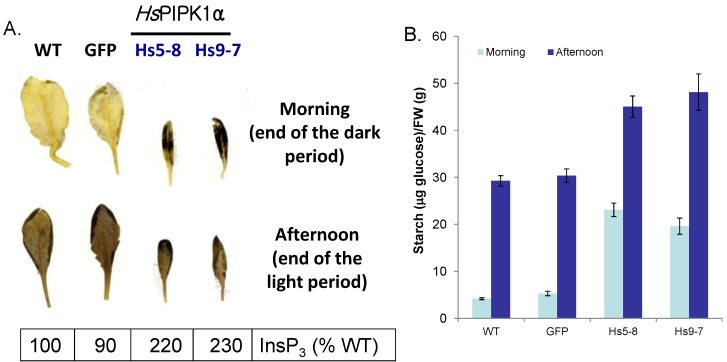
Biochemical and cellular analyses of starch indicated elevated levels of starch in *Hs*PIPKIα plants. (**A**) Leaves were harvested at the end of dark period (Morning, prior to lights coming on) or end of light period (Afternoon, prior to lights going off). Chlorophyll was removed with hot ethanol (80% [v/v]) extraction and starch was stained with 1% Iodine solution (I_2_/KI) and photographed with a Nikon CoolPix 4500 camera. Relative InsP_3_ by mass measurement is shown (WT = 100%); (**B**) Short day (8h light/16 h dark) cycle grown 3 week-old seedlings were harvested at the end of dark period (Morning) and the end of light period (Afternoon) and boiled in ethanol. Starch in the ethanol-insoluble fraction was measured after enzymatic digestion with α-amylase and amyloglucosidase to make glucose. The data are the mean of duplicate ± SD from 3 independent experiments.

Previously, we showed that increasing the flux through the PI pathway in tobacco cells grown in suspension culture resulted in increased sucrose uptake, increased respiration and with time, increased starch granules [49,59]. To visualize starch in *Hs*PIPKIα plants, leaves from 6 week-old plants were stained with iodine (Figure 8A). Leaves from all the lines had less starch in the morning (morning is defined as plants harvested in the dark at 9 AM, 1 h before the lights came on) than afternoon (plants harvested at 5 PM, 1 h before the lights went off); however, leaves from *Hs*PIPKIα plants showed significantly more starch than wild type. The increased starch was evident in leaves harvested both in the morning and afternoon. To quantify the differences, starch was analyzed from leaves of 3-week-old seedlings. In the *Hs*PIPKIα leaves the starch was 5-fold higher in the morning samples and 1.5-fold higher in the afternoon samples compared to WT and GFP (Figure 8B). Since excessive starch accumulation can result in changes in chloroplast morphology, chloroplast structure was compared using EM. The chloroplasts of the *Hs*PIPKIα plants appeared normal although swollen because of the large starch granules (Supplementary Figure 1) and total chlorophyll was not different in any of the lines (Supplementary Figure 2). 

**Figure 9:**
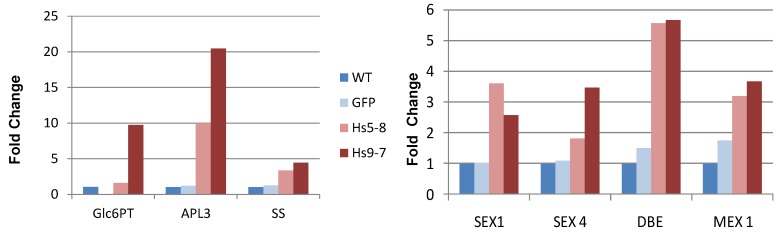
QRT-PCR was carried out using Full Velocity SYBR Green PCR Master Mix (Stratagene) and with the primers for genes that are involved in starch metabolism. The transcript level of each gene monitored is expressed as the fold change compared to the level of expression in the WT. The raw data (Ct values) were normalized using actin or PP2A as an internal control. The experiment was reproduced twice with similar results. Representative data are shown. Abbreviations: Glc6PT (glucose-6-phosphate transporter), APL3 (glucose-1-phosphate adenylyltransferase/ADP-glucose pyrophosphorylase), SS (starchsynthase), SEX (starch excess proteins), SEX1 (glucan water dikinase), SEX4 (phosphoglucan phosphatase), DBE (debranching enzyme, α-amylase activity), MEX (maltose transporter).

To further investigate how starch metabolism is altered in *Hs*PIPKIα plants at the molecular level we monitored transcripts levels of genes that are involved in starch synthesis [60,61]. The ADP-glucose pyrophosphorylase 3 (APL3), which converts G1P and ATP to ADP-glucose and starch synthase (SS), responsible for elongating starch polymers, were up-regulated in both lines of the *Hs*PIPKIα plants compared to WT and GFP (Figure 9A). The glucose-6-phosphate transporter (Glc6PT), which imports Glc6P from the cytosol into the chloroplast where it is converted to glucose-1-phosphate, was up-regulated in the Hs9-7 line but was only marginally increased (1.6 fold) in the Hs5-8 line. We also monitored transcript levels of genes that are involved in starch degradation such as glucan water dikinase (SEX1), phosphoglucan phosphatase (SEX4), α-amylase (DBE) and maltose transporter (MEX). They were all highly expressed in *Hs*PIPKIα plants compared to WT and GFP plants (Figure 9B). Although starch degradation genes were higher in the kinase plants, the relative loss of starch during the night was only 50%–60% in the *Hs*PIPKIα plants whereas 85%–87% of the starch was lost in the WT and GFP plants. These data suggest that the *Hs*PIPKIα plants were not mobilizing all the starch during the night to sustain cellular metabolism. Light/dark regulation of starch metabolism is complex. While starch metabolism appears to be under circadian control [62], plants are able to respond to environmental cues and adjust starch metabolism to compensate for day length [63]. Our data suggest that expression of *Hs*PIPKIα affected the light/dark sensing that regulates starch metabolism.

If carbon export from the leaves to root was affected, the increase in starch might have been accompanied by an increase in sucrose. We did not detect significant differences in soluble sugars in the *Hs*PIPKIα 5-8 (Hs5-8) seedlings although there was a slight increase in sucrose in leaves of 3 week-old *Hs*PIPKIα 9-7 (Hs9-7) seedlings growing on agar supplemented with 1% sucrose (Supplementary Figure 3A). If sucrose was limiting in the roots, we reasoned that adding sucrose would increase root growth. When seedlings were grown on agar with increased (3%) sucrose, root growth increased slightly (Supplementary Figure 3B). Although root growth was less inhibited at 6% sucrose in the *Hs*PIPKIα seedlings, sucrose alone did not restore normal root growth. 

**Figure 10:**
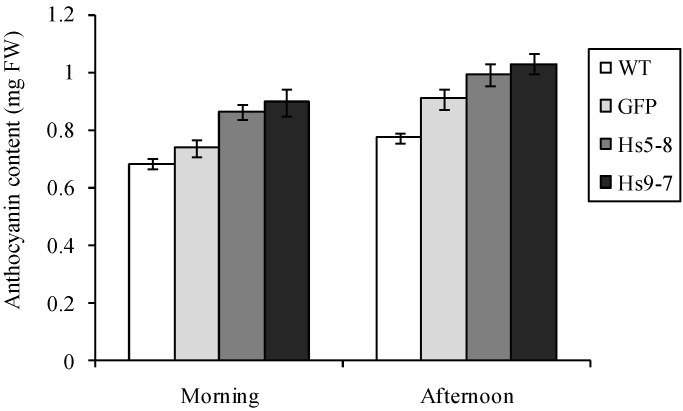
There was a 20% increase in anthocyanin in the *Hs*PIPKIα lines. Three week-old seedlings were harvested and anthocyanin was extracted and quantified as described in the Experimental Section. The data are the means ± SD of 3 biological replicates.

Increased anthocyanin biosynthesis is an indication of stress and a change in carbon flux. When anthocyanin levels were compared, the *Hs*PIPK1α plants had more anthocyanin whether they were harvested morning or afternoon (Figure 10). 

### 2.5. Constitutively Increasing PtdInsP_2_ Biosynthesis and InsP_3_ in Leaves did not Affect Photosynthetic Electron Transport

In response to changes in the environment and demands on energy and reductive power in the chloroplasts, plants can switch between cyclic and linear electron flow pathways [64]. Cyclic electron flow around photosystem I (CEF) also increases upon drought stress [65,66] and during light activation after a prolonged dark period [67]. Over the course of these experiments, we noticed that in leaves of the *Hs*PIPKIα plants, the Ins(1,4,5)P_3_ was about 2-fold higher in the afternoon compared to morning. In addition, others had reported that InsP_3_ was higher in light than dark grown plants [12]. Furthermore, previous reports indicated that expressing *Hs*PIPKIα in tobacco cells increased activity of ATP-dependent pumps and affected K^+^ channels [49,68]. We reasoned that increasing biosynthesis of PtdInsP_2_ should increase the demand for ATP and could potentially lead to activation of CEF. 

**Figure 11:**
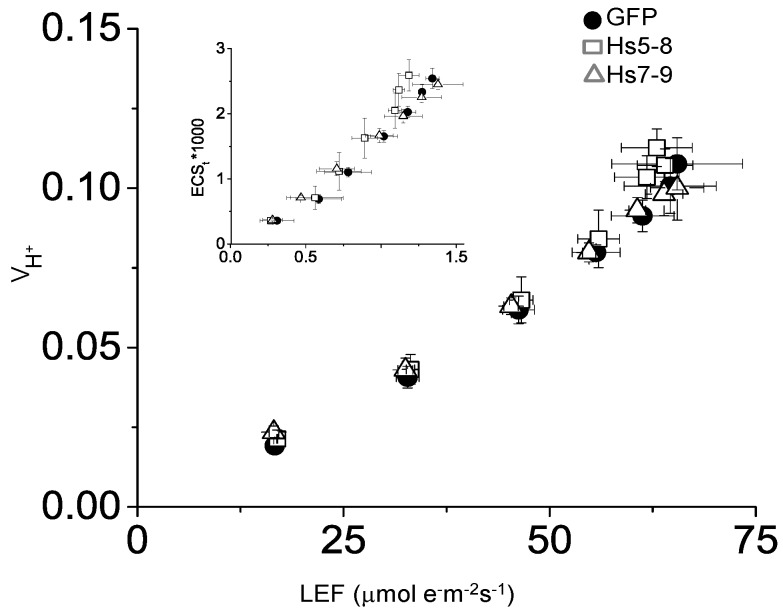
CEF is not increased in *Hs*PIPKIα plants. Light-driven transthylakoid proton flux (*v*_H_^+^) as a function of linear electron flow (LEF) rates and relative light induced *pmf*, as measured by the total amplitude of the ECS decay (ECS_t_), as a function of *pmf* attributable to LEF alone (*pmf*_LEF_) (inset). GFP (circles), *Hs*5–8 (open squares), and *Hs*9-7 (open triangles). Data represents mean and SD of individual leaves measured at increasing light intensities (60–500 μmol photons m^−^^2^ s^−^^1^, n = 3).

To investigate whether the increased PtdInsP_2_ biosynthesis reflected differences in photosynthetic electron flow, we quantified proton and electron transfer rates in 6 week-old plants to look for increased CEF. Figure 11 shows the steady state transthylakoid proton flux (*v*_H_^+^) as a function of linear electron flow (LEF) rates at multiple light intensities. The H^+^/e^−^ for LEF is fixed, and any increase in the slope of the *v*_H_^+^/LEF relationship would indicate an increase in proton translocation independent of LEF due to the activation of CEF [69]. Figure 11 shows no statistically significant increase in this slope (ANCOVA *p* > 0.05, n = 3) for either of the *Hs*PIPKIα plants, suggesting no activation of CEF due to our calculated increase in ATP demand. This absence of CEF is further evidenced by comparison of *pmf* (expressed as total amplitude of electrochromic shift (ECS) during a dark interval (ECS_t_)) to *pmf* attributable to LEF alone (*pmf*_LEF_) [69,70]. An increase in CEF should cause an upwards shift in the relationship of these parameters; however, we see no significant differences in either of the *Hs*PIPKIα plants when compared to GFP (ANCOVA *p* > 0.05, n = 3). Taken together, Figure 9, Figure 10 and Figure 11 clearly show that the constitutive increase in the PI pathway affected chloroplast carbon metabolism and transcripts involved in starch biosynthesis while having little impact on photosynthetic electron transport.

The data in Figure 11 show that any increase in demand for ATP imposed by expressing *Hs*PIPKIα was not met by increasing cyclic electron flux. There was no significant difference in the total ATP recovered from the *Hs*PIPKIα and WT seedlings (Supplementary Figure 4A) and analysis of the NADPH/NADP ratio indicated that it was slightly less in the *Hs*PIPKIα plants. The NADPH/NADP ratio in seedlings was 2.5 ± 0.07, 1.9 ± 0.15, 0.9 ± 0.24 and 0.9 ± 0.24 for the WT, GFP and *Hs*5-8 and *Hs*9-7 seedlings, respectively. (The NADPH value for the WT seedling was 2.7 μmol/g FW and for NADP was 1.0 μmol/g FW). The NADPH/NADP ratios for leaves of whole plants were 1.2, 0.8, and 0.5 for WT, *Hs*5-8 and *Hs*9-7, respectively. Based on these observations, it is likely that in order to maintain homeostasis there were changes in metabolic pathways (e.g., an increase in the malate s huttle [64,71] or increased mitochondrial respiration as reported for tobacco cells [49]) that provided any additional ATP needed as a result of the expression of *Hs*PIPKIα. 

### 2.6. Physiological Characteristics of the HsPIPK1α Plants

Although one might reason that a constitutive InsP_3_-mediated signal would make the plants stress tolerant, it was also possible that stress-induced changes in basal metabolism would render the plants stress sensitive [52]. Specifically, the constitutive increase in InsP_3_-mediated signaling should deplete InsP_3_-sensitive calcium stores of the organelles and render the remaining calcium tightly bound. If this were true, then the total cellular calcium would be reduced and stress responses that require an increase in stored (organelle) calcium might be compromised. As shown in Table 2 above, there was a 10% decrease in overall calcium in the *Hs*PIPKIα plants. We used several approaches to test for stress tolerance. 

**Figure 12:**
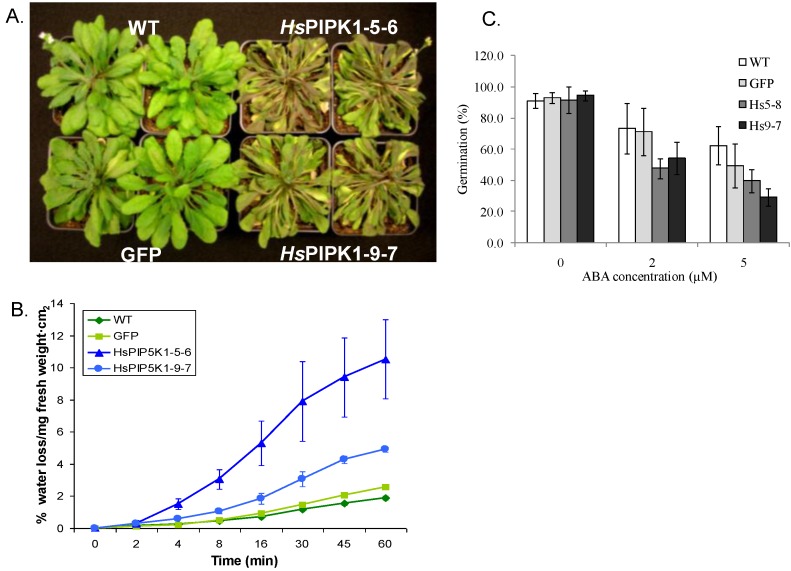
The *Hs*PIPKIα plants are drought sensitive and have increased water loss in the detached leaf assay. Plants were grown under a short day cycle (8 h of light/16 h of dark) at 21 °C with a light intensity of 150 µmol·m^−2^·s^−1^ in the North Carolina State University Phytotron in a growth chamber. (**A**) Water was withheld for 9 days and plants were photographed; (**B**) Detached leaves from well-watered plants were used to measure water loss over time; (**C**) The seeds from the *Hs*PIPKIα lines are more sensitive to ABA. The data are evaluated 3 days after seed germination on media containing different concentration of ABA. ~100 seeds per each concentration were tested in 3 independent experiments (Mean ± SE).

Perera *et al*. [15] showed that plants with constitutively low InsP_3_ had increased tolerance to the withdrawal of water for up to 12 days. The authors concluded that the plants with constitutively low InsP_3_-mediated signaling had induced compensatory pathways that rendered the plants drought tolerant. To test the drought tolerance of the *Hs*PIPKIα plants, we withheld water for 9 days. As shown in Figure 12A, the *Hs*PIPKIα plants were more drought sensitive than WT plants. In addition, the *Hs*PIPKIα plants had increased leaf water loss in a detached leaf assay (Figure 12B). The phenotype is the opposite of the plants with low InsP_3_ reported by Perera *et al*. [15]. The data could be interpreted as indicating that InsP_3_-mediated responses were not involved in stomatal closure. However, it is possible that a decrease in organelle calcium stores or extracellular calcium affected the ability of the guard cells to close. We did not measure extracellular calcium per se, but InsP_3_ has been shown to increase in response to added extracellular calcium, and this response requires the presence of the chloroplast thylakoid calcium binding protein, CAS [72,73]. If the guard cells in the *Hs*PIPKIα plants had depleted chloroplast calcium stores or extracellular calcium, in theory, the stomata should not have closed as rapidly and the plants should be more sensitive to water loss. It is also possible by increasing the flux through the PI pathway and increasing PtdInsP_2_ but decreasing PtdIns4P, we affected membrane biogenesis and/or plasma membrane pumps and channels such that stomatal closure was impaired and the plants wilted faster [14,16,68]. More extensive studies of guard cell calcium stores and membrane trafficking are needed to determine the underlying mechanisms rendering the *Hs*PIPKIα plants drought sensitive. It should be noted that this phenotype of the *Hs*PIPKIα plants is in contrast to what was reported for the sac9 (PtdInsP_2_ ptase) mutants which have increased PtdInsP_2_. The sac9 mutants were reported to have constitutively closed stomata [46]. These differences in the phenotype of the sac9 mutant and *Hs*PIPKIα plants may reflect differences in the levels of PtdInsP_2_ in the leaves of the sac9 and *Hs*PIPKIα or other effects of the sac9 mutation that may have more direct effects on membrane biogenesis and cell wall deposition [74].

Several labs have reported changes in InsP_3_ in response to abscisic acid (ABA); however, these genetic approaches to increase or decrease InsP_3_ by altering the expression of phospholipase C or InsP ptases have had mixed results [28]. Ectopic expression of endogenous InsP Ptase1 to lower InsP_3_ decreased ABA-induced stomatal closure, while lowering InsP_3_ by expressing the human InsP 5-ptase increased the ABA-sensitive stomatal closure. The phenotypes of the loss of function mutants are more consistent. Mutations in InsP Ptase1 and 2 resulted in increased InsP_3_ and increased sensitivity to ABA in seed germination assays [75], and plants with a mutation in InsP ptase12, an InsP ptase [76] have pollen that germinates precociously and are hypersensitive to ABA [77]. As predicted from these mutants, the *Hs*PIPKIα seeds germinated quickly and were more sensitive to ABA than wild type in germination assays (Figure 12C). The *Hs*PIPKIα seedlings were also similar to the InsP ptase mutants in that they had an incomplete venation pattern in the cotyledons [78] (data not shown). 

**Figure 13:**
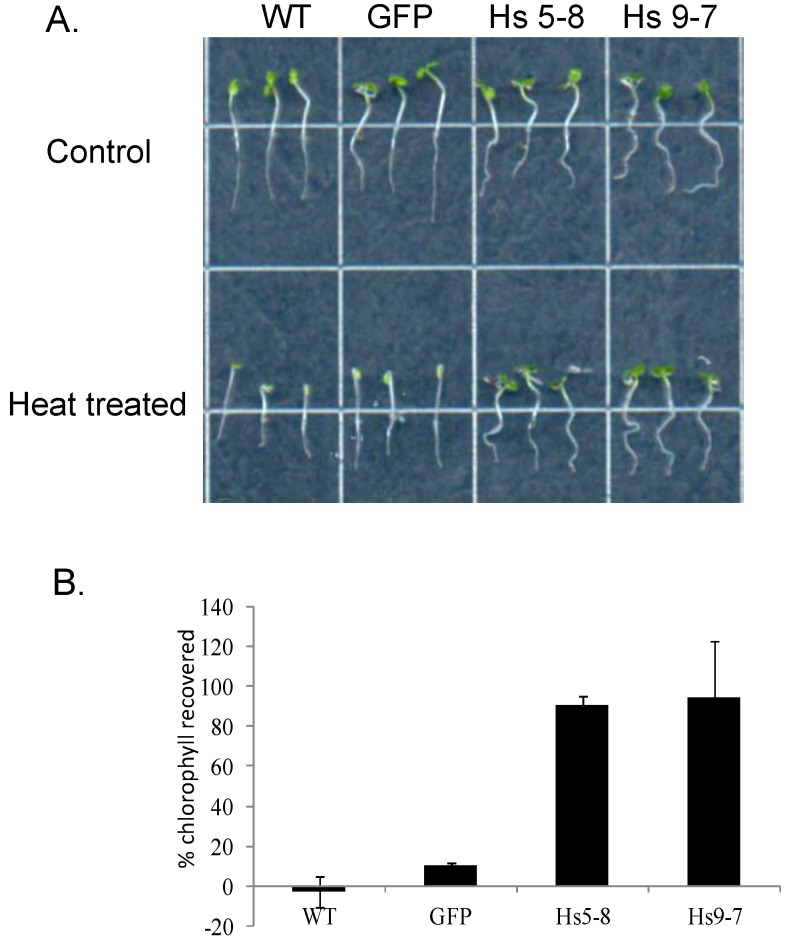
*Hs*PIPKIα seedlings are more tolerant of heat and light. (**A**) Seedlings were grown in the dark for 2.5 days, exposed to 48 °C for 30 min and placed in the light 24 h or kept in the light at room temperature (control); (**B**) Chlorophyll was extracted in acetone and absorbance was measured at 663 nm. The data are reported as % of the control chlorophyll recovered per g FW. The data are the means ± SD for 3 biological replicates consisting of 25 seedlings.

Regulation of cytosolic calcium is important for heat tolerance [79,80,81] and heat has been shown to increase PtdInsP kinase activity and PtdInsP_2_ in tobacco cells [82]. We asked whether the *Hs*PIPKIα, which have increased PtdInsP_2_ would be heat tolerant. The *Hs*PIPKIα seedlings were grown in the dark for 2.5 days, exposed to 48 °C for 30 min and then placed in the light for 24 h. As shown in Figure 13, the *Hs*PIPKIα seedlings were more heat tolerant. Survival was quantified by measuring chlorophyll recovery after 24 h. 

### 2.7. Very Few Differences in Transcript Levels Were Detected Using Microarray Analysis

In an attempt to gain some insight into what affects expressing *Hs*PIPKIα had on plant gene expression, we did a microarray analysis of cDNA from three-week-old seedlings harvested just before the lights came on (morning). Table 3 reveals the results of the analysis of both *Hs*PIPKIα 9-7 and 5-8 lines compared to the WT controls. Supplementary Figure 4B shows a heat map of changes detailed in Table 3. Some of the transcript changes may reflect systemic changes in vascular transport and cell wall structure associated with up-regulation of the PI pathway [51,74,83,84,85]. Transcripts of PIPKs were first reported associated with vasculature [51,86] and these transcript changes may reflect tissue specific sensitivity to the expression of the *Hs*PIPKIα or a reflection of effects on long distance signaling by InsP_3_-mediated events [87]. In addition, the transcript changes may reflect an up-regulation of pathogen responses or endocytic pathways associated with changes in phosphoinositides induced during symbiosis (e.g., PR1, Thioredoxin h8 [18,19,88,89]). We did not detect significant changes in the starch biosynthetic transcripts in the array. This may be due to differences in sensitivity of the standard microarrays compared to qPCR. Additional studies of tissue specific, targeted gene expression, cell wall structure and pathogen response are necessary to understand the impact of increased flux through the PI pathway induced in these studies. 

**Table 3:** Genes with greater than two-fold change in expression in the *Hs*PIPKIα 9-7 and 5-8 lines compared to WT. Notations in parentheses indicate the specific line (9-7 or 5-8).

AGI Locus ID	Gene Descriptor	Microarray Fold Change	Log Ratio
**Up-regulated Expression**			
At2g14610	pathogenesis-related protein 1 (PR-1)	10.97 (9-7)	3.46 (9-7)
		2.40 (5-8)	1.26 (5-8)
At3g15650	phospholipase/carboxylesterase family protein	5.56 (9-7)	2.48 (9-7)
		4.32 (5-8)	2.11 (5-8)
At1g73040	jacalin lectin family protein	5.36 (9-7)	2.42 (9-7)
		4.48 (5-8)	2.16 (5-8)
At1g69880	thioredoxin, putative	4.91 (9-7)	2.30 (9-7)
		3.69 (5-8)	1.88 (5-8)
At1g19960	transmembrane receptor, putative	4.27 (9-7)	2.09 (9-7)
		2.73 (5-8)	1.45 (5-8)
At4g23680	major latex protein-related	3.38 (9-7)	1.76 (9-7)
		3.11 (5-8)	1.64 (5-8)
At1g32450	proton-dependent oligopeptide transport (POT) family protein	3.22 (9-7)	1.69 (9-7)
		2.18 (5-8)	1.13 (5-8)
At4g15110	cytochrome P450 97B3, putative	2.91 (9-7)	1.54 (9-7)
		3.43 (5-8)	1.78 (5-8)
At4g32280	auxin-responsive family protein	2.74 (9-7)	1.45 (9-7)
		2.34 (5-8)	1.23 (5-8)
At4g12550	protease inhibitor/seed storage/lipid transfer protein (LTP) family protein	2.68 (9-7)	1.42 (9-7)
		2.70 (5-8)	1.43 (5-8)
At5g39110	germin-like protein, putative	2.48 (9-7)	1.31 (9-7)
		2.48 (5-8)	2.48 (5-8)
At5g59520	zinc transporter (ZIP2)	2.45 (9-7)	1.29 (9-7)
		2.89 (5-8)	1.53 (5-8)
At3g25830	myrcene/ocimene synthase (TPS10)	2.42 (9-7)	1.27 (5-8)
		3.69 (9-7)	1.89 (5-8)
At4g30170	peroxidase, putative	2.40 (9-7)	1.26 (9-7)
		2.00 (5-8)	1.00 (5-8)
At1g78340	glutathione S-transferase, putative	2.39 (9-7)	1.26 99-7)
		2.26 (5-8)	1.18 (5-8)
At3g28530	gypsy-like retrotransposon family	2.27 (9-7)	1.18 (9-7)
		2.00 (5-8)	1.00 (5-8)
At3g62040	haloacid dehalogenase-like hydrolase family protein	2.22 (9-7)	1.15 (9-7)
		3.03 (5-8)	1.60 (5-8)
At3g08860	alanine-glyoxylate aminotransferase	2.17 (9-7)	1.12 (9-7)
		2.50 (5-8)	1.32 (5-8)
At2g01520	major latex protein-related	2.10 (9-7)	1.07 (9-7)
		2.18 (5-8)	1.12 (5-8)
At3g46130	myb family transcription factor (MYB48)	2.03 (9-7)	1.02 (9-7)
		2.40 (5-8)	1.26 (5-8)
At3g49160	pyruvate kinase family protein	2.00 (9-7)	1.00 (9-7)
		3.16 (5-8)	1.66 (5-8)
**Down-regulated Expression**			
At3g22640	cupin family protein	−2.08 (9-7)	−1.06 (9-7)
		−2.48 (5-8)	−1.31 (5-8)
At5g14180	lipase family protein	−2.40 (9-7)	−1.27 (9-7)
		−2.27 (5-8)	−1.19 (5-8)
At2g34600	jasmonate-zim-domain protein 7	−2.34 (9-7)	−1.23 (9-7)
		−2.28 (5-8)	−1.19 (5-8)
At1g66900	α/β-hydrolase domain-containing Protein	−2.65 (9-7)	−1.41 (9-7)
		−2.27 (5-8)	−1.18 (5-8)

* All reported fold changes have *p* values <0.05.

## 3. Experimental

### 3.1. Generation and Selection of HsPIPKIα Transgenic Plants

The gene encoding the human PIPKIα (NM_003557) was cloned into pK7WGF2 (Functional Genomics Division, Department of Plant Systems Biology, Ghent University, Ghent, Belgium as previously described [49]. Recombinant plasmids were transformed into *Agrobacterium tumefaciens* EHA105 using the freeze-thaw method [90] and then transformed into *Arabidopsis* (*Arabidopsis thaliana* ecotype Columbia) by the floral dip method [91]. Four independent transformed lines were further selected. Stable expression of the transgene was monitored by RT-PCR and immunoblotting as described below.

### 3.2. Plant Growth Conditions

Wild-type (ecotype Columbia) and *Hs*PIPKIα transgenic *Arabidopsis thaliana* plants were grown under short-day conditions (8 h of light/16 h of dark) at 21 °C with a light intensity of ~150 µmol·m^–2^·s^–1^ in the North Carolina State University Phytotron in a growth chamber. For all soil-grown experiments, a large batch of soil mix (Promix PGX; Hummert International, Earth City, MO, USA) was moistened well with water and the pots were filled with an equal amount of soil prior to planting the seeds. For experiments using seedlings, seeds were surface-sterilized by first incubating in 70% ethanol for 1 min, then incubating in a mixture of 30% (v/v) commercial bleach and 0.1% Triton X-100, with occasional agitation for 12 min and then washed with sterilized dH_2_O for 7 times and stratified for 48 h at 4 °C prior to plating on Murashige and Skoog medium (Caisson Labs, North Logan, UT, USA) containing 1% sucrose and 0.8% agar type M (Sigma-Aldrich, St Louis, MO, USA). Plates were incubated vertically in a growth chamber under short-day conditions as described above. For root and hypocotyl elongation measurements, 4 day after germination plates were covered and placed in the dark and growth was monitored every 24 h for a 3- to 4-day period.

### 3.3. Seed Germination Assays

Surface-sterilized, stratified seeds were plated on Murashige and Skoog plates containing different concentrations of ABA as indicated. Germination was counted as the emergence of green cotyledons at 3 days after plating.

### 3.4. RNA Extraction, RT-PCR, and qRT-PCR Analysis

RNA was isolated from harvested leaves using the plant RNeasy Mini kit (Qiagen Sciences Inc., Frederick, MD, USA) with the on-column RNase-free DNase I treatment. RT was carried out to generate cDNA using Omniscript reverse transcriptase enzyme (Qiagen Sciences Inc.) and random primers according to the manufacturer’s instructions (Qiagen Sciences Inc.). For RT-PCR, cDNAs were amplified using HotStar Taq DNA Polymerase (Qiagen Sciences Inc.) and gene-specific primers. q RT-PCR was carried out using Full Velocity SYBR-Green QPCR Master Mix (Stratagene, La Jolla, CA, USA) on an MX3000P thermocycler (Stratagene). Gene-specific primers for select genes were designed with the help of AtRTPrimer, a database for generating specific RT-PCR primer pairs [92], and are shown in Supplementary Table 1. PCR was optimized, and reactions were performed in duplicate. Transcript levels were standardized based on cDNA amplification of the reference gene *ACTIN2/8* and/or *PP2A*. Relative gene expression data were generated using the 2^–^ΔΔ^Ct^ method [93] using the wild-type as the reference. 

### 3.5. Protein Isolation and Immunoblotting

Total protein extract was obtained from plants frozen in liquid N_2_ or seedlings grown as described by Weigel and Glazebrook [94]. Microsomal fraction proteins were obtained by two-phase partitioning as described previously [49]. Protein concentrations were quantified as described by Bradford [95]. Protein was separated by 10% (w/v) SDS-PAGE and transferred to PVDF membrane by electroblotting and membranes were incubated with antibodies (anti-mouse GFP [Clonetech Lab, Mountain View, CA, USA]), and incubated with horseradish peroxidase-conjugated anti-mouse or anti-rabbit. Immunoreactivity was visualized by incubating the blot in SuperSignal West Pico Chemiluminescent substrate (Pierce Protein Products, Thermo Fisher Scientific, Rockford, IL, USA) and exposure to X-ray film. After chemiluminescence detection, total protein was visualized by staining the blots with Amido black (Sigma-Aldrich, St Louis, MO, USA). Following chemiluminescence detection, total protein was visualized by staining the blots with Amido black (Sigma-Aldrich, St Louis, MO, USA).

### 3.6. PtdInsP 5-Kinase Assays

*In vitro* lipid kinase assays were performed using plasma membrane proteins (2 µg) and endomembrane fraction protein (30 µg). The standard assay was as previously described [49] with the following modifications. Reactions were performed either in the absence or presence of substrate 125 µM PtdIns(4)P from porcine brain (Avanti Polar Lipids) at room temperature for 10 min in a total volume of 50 µL. After incubation, phospholipids were extracted and separated by TLC as described [96]. 

### 3.7. Ins(1,4,5)P_3_ Assays

Seedlings (17-day-old) and leaves from 1 month-old plants were harvested immediately, frozen in liquid N_2_, ground to a fine powder, and precipitated with cold 10% (v/v) perchloric acid (PCA). Ins(1,4,5)P_3_ assays were performed using the TRK1000 Ins(1,4,5)P_3_ assay kit (Amersham Pharmacia Biotech, Piscataway, NJ, USA) according to the manufacturer’s instructions.

### 3.8. Lipid Profiling

To determine the effects of *Hs*PIPKIα expression on total glycerol lipid profile, we extracted lipids from leaves from 3 week-old seedlings in the protocol as described by the Kansas Lipidomics Facility [97] and lipid analysis and quantification were performed as described [49] at the Kansas Lipidomics Facility. 

### 3.9. *In Vivo* Labeling Studies

For short-term labeling studies with ^32^Pi, 13 or 17-day-old seedlings (~10 seedlings per well) were transferred to a multi-well plate containing 800 µL of 0.5× Murashige and Skoog medium. The seedlings were incubated overnight with gentle rotation. In the morning, 50 µCi of carrier-free [^32^P] Pi (~62 µCi mL^–1^) was added to each well and seedlings were harvested at the indicated time points by immediate transfer to 500 µL of cold 20% (v/v) PCA and incubated on ice for ~20 min. The PCA treated seedlings were then washed with cold water twice, and lipids were extracted, separated by TLC, and ^32^P-labeled lipids were quantified with a Bioscan Imaging Scanner. 

### 3.10. Labeling Studies with [^3^H]*myo*-Inositol

One-week-old seedlings (~10 seedlings per well) were transferred to a multiwell plate containing 800 µL of 0.5× Murashige and Skoog medium containing 45 µCi of [^3^H]*myo*-inositol. Plates were incubated in a growth chamber under long-day conditions with gentle rotation to ensure aeration for 4 days. After incubation, the seedlings were quickly blotted on tissue and ground in liquid N_2_. The frozen ground powder was incubated in 0.75 N HCl containing 0.2% phytate (as carrier) on ice for 20 min. The pellet and supernatant were separated by centrifugation, the pellet was washed with cold water twice, and the [^3^H] *myo*-inositol labeled lipids were extracted from the pellet. The lipids were separated by TLC and quantified with a Bioscan Imaging Scanner. [^3^H] inositol hexaphosphate was also identified from the supernatant based on the coelution of standard Ins(1,2,3,4,5,6)P_6_ using ion chromatography. For these analyses, fifty microliters of the HCL extract were diluted to 1 mL with 0.375 N HCl and filtered through a 0.45 μm nylon filter. Fifty and one hundred microliter aliquots were analyzed by isocratic ion chromatography using 0.25 N HNO_3_ eluant and Dionex AG7/AS7 columns as previously described [98,99]. Twelve 1 mL fractions were collected at 1 min intervals and counted with 5 mL EcoLume in plastic scintillation vials. InsP_6_ was calculated as the cpm in fraction 8 divided by the total cpm of the 12 fractions times 100%. Two biological replicates were analyzed to give a total of two wild-type and two *Hs*PIPKIα line extracts. Two analyses (50 μL and 100 μL) were performed on each of the four diluted extracts.

### 3.11. Determination of Total InsP_6_ in Seeds

InsP_6_ analysis of seeds was a modification of a previous method described by Bentsink *et al*. [100]. Specifically, dry seeds (4–5 mg) were rehydrated in 500 μL 0.5 N HCl for 60 min at 55 °C. The mixture was ground with a plastic pestle and centrifuged 5 min at 15,000 ×*g*. The supernatant solutions were filtered through a 0.45 μm pore size 17 mm nylon filter, diluted with an equal volume of water, and InsP_6_ was determined by isocratic ion chromatography using 0.25 N HNO_3_ eluant and Dionex AG7/AS7 columns as previously described [98,99]. Triplicate biological samples were analyzed.

### 3.12. Quantification of Soluble Pi

Leaves of 3-week-old seedlings were harvested, immediately frozen in liquid N_2_, and ground to a fine powder. Soluble Pi was extracted by adding 10 times 1% [v/v] HOAC of sample weight. The extracted sample was analyzed for Pi as described by Bartlett [101] measuring A660.

### 3.13. Determination of Anthocyanin and Chlorophyll A

Anthocyanin content was determined as describe in Teng *et al.* [102]. Frozen samples from 3 week-old seedlings were homogenized in 1% [v/v] HCl in MeOH at 4 °C and incubated overnight. After centrifugation at 15,000 ×*g* for 15 min, the absorbance of supernatants was measured at 530 and 657 nm and anthocyanin was calculated using the formula A_530_ − 0.25 × A_657_ and corrected for the volume and sample weight. 

For chlorophyll a measurements, the samples (25 seedlings/treatment) were extracted in ethanol (100% v/v). Chlorophyll was quantified by measuring the absorbance at 665 nm (Eb665) and 750 nm (Eb750). After the reading, the samples were acidified by adding 10 μL of 2N HCl directly to the cuvette, mixed well, incubated for 5 min, read at 665 nm (Ea665) and 750 nm (Ea750). Chlorophyll was calculated using the formula 29.6 × [(Eb_665_ − Eb_750_) − (Ea_665_ −E a_750_)] and reported as % of the control (non-heat treated samples) for each line or per g FW as indicated.

### 3.14. Staining and Quantification of Starch

For starch staining, leaves were harvested from 6 week-old plants at the end of the day and at the end of the night. Chlorophyll was removed with 80% EtOH and stained with IKI solution (1% [w/v] iodine, 2% [w/v] potassium iodine) for 1 min and rinsed with dH_2_O and imaged by scanner. For starch quantification, frozen samples from 3 week-old seedlings were homogenized in 80% (v/v) EtOH and boiled for 3 min and centrifuged at 3,000 ×*g* for 10 min. Insoluble fraction was determined by measuring the amount of glucose released by treatment with α-amylase and amyloglucosidase, as described by Smith and Zeeman [103]. 

### 3.15. Analysis of ATP and NADP(H) and NAD(H)

ATP was assayed using a bioluminescence assay kit (Sigma-Aldrich) according to the manufacturer’s directions. NADP(H) and NAD(H) were extracted and assayed as described by Matsumura and Miyachi using an enzyme cycling assay measuring the absorbance at 570 nm [104]. 

### 3.16. Sugar Analysis

Soluble sugars and inositol were analyzed by gas chromatography-mass spectrometry. Leaf tissue of 3 week-old seedlings was ground in a cold 60:40 (v/v) methanol:H_2_O solution, mixed with acetonitrile, and dried under vacuum. Samples were analyzed at the Metabolomics and Proteomics Laboratory at North Carolina State University. The sugars were converted to trimethylsilyl derivatives, and gas chromatography-mass spectrometry was performed using a ThermoTrace GC Ultra gas chromatograph coupled to a Thermo DSQ II mass spectrometer. The mass spectrometer was operated with an electron-impact source in positive mode monitoring *m/z* 191, 204, 217, 361, and 437. Quantitation was conducted by comparing peak areas obtained for trimethylsilyl derivatives of fructose, glucose, and sucrose in the samples with a series of reference standards analyzed concurrently, and data were processed using Thermo’s Xcalibur software. Data presented are averages from three independent biological replicates.

### 3.17. ICP Analysis

All elements except Cu were analyzed on a Perkin Elmer inductively coupled plasma-optical emission spectrometer (ICP-OES). 50 mg of pre-weighted dried leaves of 3 week-old seedlings were digested with 4 mL of conc. HNO_3_ (Trace Metal Grade) and 2 mL of 30% H_2_O_2_ (ACS reagent grade). Due to the low concentration of Cu in sample digestates (ppb), Cu was determined by inductively coupled plasma mass spectrometry using a Varian-820 Quadrupole ICP-MS.

### 3.18. *In Vivo* Spectroscopic Analysis

Photosynthetic parameters were measured using an in-house constructed spectrophotometer/fluorimeter modified from [105] with humidified air supplied to the underside side of the leaf, as described in [70,106,107,108]. LEF rates were calculated as:

*LEF* = ϕ_II_ * i * 0.4 (1)

Where ϕ_II_ is the yield of photosystem II calculated using chlorophyll a fluorescence changes from steady state to saturating light [109,110], and *i* is the actinic light intensity. 

The thylakoid proton circuit was monitored using dark interval relaxation kinetics of the electrochromic shift (ECS) of absorption at 520 nm of the carotenoids in response to the transthylakoid electric field [69]. Total light induced *pmf* was estimated as the total ECS from light to dark (ECS_t_). Light induced transthylakoid proton flux (*v*_H_^+^) was estimated from the initial slope of the ECS from light to dark. The *pmf* attributable to LEF (*pmf*_LEF_) was calculated as:

*pmf_LEF_* = *LEF* * *τ_ECS_* (2)

Where *τ_ECS_* is the lifetime of the ECS decay [69,70]. 

Data analysis was performed in, and descriptive statistics and figures were generated with Origin 9.0 (Microcal Software). Statistical analysis was performed using MATLAB R2012a (The Mathworks). Statistical significance was set at *p* < 0.05. 

### 3.19. RNA Isolation for Microarray Analysis

For the microarray analysis, leaf samples were collected from 3 week-old seedlings harvested in the dark just before the lights came on and immediately ground in liquid N_2_. Three biological replicates were performed for the wild type, GFP, and two independent transgenic lines (Hs5-8 and Hs9-7). RNA was isolated using the Plant RNeasy kit (Qiagen Sciences Inc., Frederick, MD, USA), and biotinylated target cRNA was synthesized using the 3' IVT Express kit (Affymetrix, Santa Clara, CA, USA). RNA quality was monitored on an Agilent 2100 bioanalyzer. *Arabidopsis* arrays (ATH1 from Affymetrix) were hybridized, and the data acquisition and analysis were performed by Expression Analysis using the Affymetrix fluidics station and GCOS software.

## 4. Conclusions

Leaves of plants expressing *Hs*PIPKIα had increased PtdInsP_2_ biosynthesis and increased total InsP_3_. Our focus was to characterize the effects of increasing the flux through the PI pathway in leaves. Compared to WT and GFP-expressing plants, the leaves of the *Hs*PIPKIα plants had increased starch and anthocyanin both at the end of day and end of night. InsP_3_ levels were highest in the afternoon in the HsPIPKIα plants, correlating positively with photosynthesis. Although chloroplast carbon metabolism was affected, photosynthetic electron transport was not different in the *Hs*PIPKIα plants compared to the WT or GFP controls. There are many reports indicating a role for calcium in the chloroplast and specifically for changes in stromal calcium during the light/dark transition [31,34,35,36,37]. Johnson *et al*. [36] suggested that cytosolic calcium might be the source of calcium for the stromal increase during the light/dark transition and showed that photosynthetic electron transport was not required for the dark-induced stromal calcium changes; however, the role of cytosolic calcium in regulating chloroplast and organelle metabolism remains a conundrum [40,41]. Based on previous work and the data presented in this paper, we hypothesize that InsP_3_ is one of the components of cytosolic signaling which affects chloroplast calcium homeostasis and that InsP_3_ likely contributes to coordinating organelle calcium signaling during basal metabolism, as well as light/dark transitions and stress-induced responses. While more extensive studies with tissue and organelle-specific calcium probes [111,112,113] are needed to determine whether a constitutive InsP_3_ signal can affect chloroplast calcium and or light/dark calcium fluctuations, the *Hs*PIPKIα plants, which have increased flux through the PI pathway, provide a platform for these studies.

## Supplementary Files

Supplementary File 1
